# The Bee Sting That Was Not: An Unusual Case of Hymenoptera Anaphylaxis Averted in a Patient Treated with Omalizumab for Asthma

**DOI:** 10.1155/2014/138963

**Published:** 2014-08-12

**Authors:** Evelyn M. Slaughter, Nathan Boyer, Steven Bennett

**Affiliations:** ^1^Department of Internal Medicine, Madigan Army Medical Center, 9040 Fitzsimmons Drive, Tacoma, WA 98431, USA; ^2^Department of Pulmonary and Critical Care Medicine, Brooke Army Medical Center, 3851 Roger Brooke Drive No. 3600, San Antonio, TX 78219, USA; ^3^Department of Pulmonary, Sleep and Critical Care Medicine, Madigan Army Medical Center, 9040 Fitzsimmons Drive, Tacoma, WA 98431, USA

## Abstract

This paper presents a case of hymenoptera venom anaphylaxis averted by omalizumab, a monoclonal antibody to IgE antibody. This case suggests a novel and unintentional effect of this therapy. Currently omalizumab is only FDA approved for the treatment of moderate-persistent allergic asthma. However case reports, such as ours have illustrated omalizumab's efficacy in the treatment of a myriad immunologic and allergic diseases. These outcomes have broadened the understanding of omalizumab's complex mechanism of action.

## 1. Background

Asthma affects over 300 million patients worldwide and it is estimated that 50% of severe asthma cases have a component of IgE-mediated allergic asthma [1]. Omalizumab is a recombinant humanized monoclonal antibody that binds to circulating IgE, therefore decreasing cell bound IgE and down regulating high affinity receptors on mast cells, basophils, and eosinophils [2]. Approved in 2003 for moderate to severe asthma, it is a treatment option for asthmatics not well controlled with inhaled corticosteroids and inhaled long-acting B2 agonist bronchodilators [1]. Since then there have been a few case reports of omalizumab preventing anaphylaxis due to hymenoptera venom in systemic mastocytosis and mast cell activation syndrome, as well as decreasing hypersensitivity reactions in patients undergoing venom immunotherapy (VIT) [3–5].

## 2. Case Presentation

We present a case of a 44-year-old male who was started on omalizumab in 2006 due to persistent and debilitating asthma symptoms (NHLBI step 5). He had a history of severe hypersensitivity to hymenoptera venom initially diagnosed at age twelve after an anaphylactic reaction to a witnessed insect sting, presumably vespid. At that time he was hospitalized for 48 hrs, treated with intramuscular epinephrine injections, prednisone, and antihistamines. At age 35, he suffered a second and third reaction while hiking. Both reactions manifested as angioedema of face, tongue, and throat, as well as persistent urticaria and wheezing. Both events resolved with intramuscular epinephrine injections, prednisone, antihistamines, and supportive care. Medications at the time of the second and third events included nasal mometasone, mometasone/formoterol MDI, levalbuterol MDI, montelukast, tiotropium, and simvastatin.

## 3. Investigations

Allergy work up at age twelve was unavailable, although patient reported positive skin testing for hymenoptera venom, exact species unknown. During second allergy evaluation at age 35 liver function tests and complete blood count were within normal limits and absolute eosinophil count was normal at 83 (0–450 u/cm). TSH, C3/C4 complement was also normal. Total IgE was elevated at 382 (0–114 KU/L) and skin testing positive for IgE to mountain cedar, dust mite, and cockroach antigens, although venom specific IGE testing was not repeated.

## 4. Differential Diagnosis

Hymenoptera venom hypersensitivity, idiopathic anaphylaxis, hereditary angioedema, acquired angioedema, and mast cell activation syndrome.

## 5. Outcome and Follow Up

Five years after initiation of omalizumab therapy for severe persistent asthma symptoms, the patient suffered a witnessed insect sting to right cheek, exact species unidentified. The insect sting resulted in only mild irritation at the site without any systemic symptoms or need for rescue medications.

## 6. Discussion

The lack of our patient's complete initial allergy evaluation and repeated hymenoptera venom IgE testing does weaken our case. However his pattern of reactions suggests possible hypersensitivity to hymenoptera venom and this pattern was disrupted after initiation of omalizumab therapy. Anti-IgE therapy has provided a novel approach for treatment of allergic asthma as well as prevention of hypersensitivity reactions. Since approval in 2007, multiple case reports and studies have enhanced our understanding of omalizumab's mechanism of action in a variety of disease processes.

Although anaphylaxis is a rare disease with a reported prevalence of 1%, severe systemic allergic reactions carry a high risk of fatality. Often, even after thorough allergic evaluation, no external trigger is reliably identified and the patient is diagnosed with idiopathic anaphylaxis. Currently, there are no RCT trials comparing the use of omalizumab in idiopathic anaphylaxis and much of our understanding is derived from case reports or studies of other allergic diseases.

Pathologic proliferation of mast cells and subsequent degranulation can lead to recurrent anaphylaxis in patients with systemic mastocytosis and mast cell activation syndrome. Insect venom and hymenoptera stings are often triggers. A case report by Kontou-Fili and Filis demonstrates successful use of omalizumab in a systemic mastocytosis patient with unprovoked and bee-sting provoked anaphylaxis. During treatment this patient had no episodes of unprovoked anaphylaxis, suffered three field stings and a 100 ug bee sting challenge without event [4].

In 2008, Jones et al. reported the successful use of omalizumab in the treatment of a 43 year old male with recurrent severe idiopathic anaphylaxis despite treatment with antihistamines, antileukotrienes and systemic corticosteroids [5]. Anti-IgE therapy has been shown to increase peanut tolerance in patients with food anaphylaxis [6]. Other case reports have shown the efficacy of omalizumab in treatment of fire ant anaphylaxis in patient who had previously failed immunotherapy [7]. Interestingly, these studies report variable effects on patients' tryptase and total IgE levels, even with significant symptomatic improvement. As elevated total tryptase levels generally reflects increased burden of mast cells, the minimal change in tryptase levels suggests that rather than a true decrease in mast cell numbers, omalizumab may increase the mast cell activation threshold by the downregulation of FcERI receptors on mast cells, basophils and, antigen-presenting dendritic cells [3, 8].

An interesting area of research has been on the effect of omalizumab in moderate to persistent asthmatics with comorbid allergic rhinitis. These are often the patients who would most benefit from immunotherapy, but also who are at higher risk for a hypersensitivity reaction from immunotherapy. Two double blind randomly controlled trials showed that when compared to immunotherapy alone, omalizumab combined with specific immunotherapy (SIT) reduced symptom severity, decreased beta-agonist use and decreased rate of respiratory reaction to SIT in allergic asthmatics with concomitant seasonal allergic rhinitis [3, 8]. These findings support the use of anti-IgE therapy in patients with severe multisystem allergic disease.

Recently in a multicenter DBRCT trial of 323 patients, three monthly injections of omalizumab had a significant dose-dependent effect on chronic urticaria and spontaneous urticaria refractory to oral antihistamines [9, 10]. Additionally, Ivyanskiy et al. reported a case series on the use of omalizumab therapy for the treatment of chronic urticaria in 19 patients. Despite varying levels of total IGE from normal to high, 84% of patients had moderate to complete resolution of symptoms. Although anti-IGE mechanisms are thought to be omalizumab's predominant mode of action, these findings provide further support that omalizumab has more than just anti-IGE effects [11, 12]. Moreover given that the majority of patients who developed anaphylaxis have urticaria as part of their reaction, these findings support the use of omalizumab in treatment of anaphylaxis.

The emerging literature on novel effects of anti-IgE therapy illustrates omalizumab's diverse effects on immune regulation. Postulated secondary effects include induction of eosinophil apoptosis, downregulation of inflammatory cytokines IL-2, IL-4, IL-13, and TNF-alpha, increase in the activity of CD4+ cells by ATP release, and decrease in basophil degranulation and downregulation of FceRI receptor expression. We hypothesize that these stabilizing effects on the immune system confer a protective effect on the immune response to exposure to insect venom as reported in our patient above. Although currently only FDA approved for treatment of allergic asthma, further research into these effects show promise for the use this anti-IGE monoclonal antibody in other atopic and immunologic diseases.

## 7. Learning Points

- Omalizumab is a monoclonal antibody which binds to circulating IgE.
- It has been proven to be highly effective in the treatment of moderate persistent allergic asthma.
- Although still under investigation, case reports have shown promise in the treatment anaphylaxis.
